# Plasmon-Assisted Direction- and Polarization-Sensitive Organic Thin-Film Detector

**DOI:** 10.3390/nano10091866

**Published:** 2020-09-17

**Authors:** Michael J. Haslinger, Dmitry Sivun, Hannes Pöhl, Battulga Munkhbat, Michael Mühlberger, Thomas A. Klar, Markus C. Scharber, Calin Hrelescu

**Affiliations:** 1 PROFACTOR GmbH, Functional Surfaces and Nanostructures, 4407 Steyr-Gleink, Austria; michael.muehlberger@profactor.at; 2 Institute of Applied Physics, Johannes Kepler University, 4040 Linz, Austria; Dmitry.Sivun@fh-linz.at (D.S.); hannes.poehl@protonmail.com (H.P.); battulga@chalmers.se (B.M.); thomas.klar@jku.at (T.A.K.); HRELESCC@tcd.ie (C.H.); 3 School of Medical Engineering and Applied Social Sciences, University of Applied Sciences Upper Austria, Garnisonstraße 21, 4020 Linz, Austria; 4 Department of Physics, Chalmers University of Technology, 41296 Göteborg, Sweden; 5 Linz Institute for Organic Solar Cells/Institute of Physical Chemistry, Johannes Kepler University, 4040 Linz, Austria; markus_clark.scharber@jku.at; 6 School of Physics and CRANN, Trinity College Dublin, Dublin, Ireland

**Keywords:** plasmons, Bragg SPPs, angle of incidence, nanoimprint lithography, grating, organic solar cell

## Abstract

Utilizing Bragg surface plasmon polaritons (SPPs) on metal nanostructures for the use in optical devices has been intensively investigated in recent years. Here, we demonstrate the integration of nanostructured metal electrodes into an ITO-free thin film bulk heterojunction organic solar cell, by direct fabrication on a nanoimprinted substrate. The nanostructured device shows interesting optical and electrical behavior, depending on angle and polarization of incidence and the side of excitation. Remarkably, for incidence through the top electrode, a dependency on linear polarization and angle of incidence can be observed. We show that these peculiar characteristics can be attributed to the excitation of dispersive and non-dispersive Bragg SPPs on the metal–dielectric interface on the top electrode and compare it with incidence through the bottom electrode. Furthermore, the optical and electrical response can be controlled by the organic photoactive material, the nanostructures, the materials used for the electrodes and the epoxy encapsulation. Our device can be used as a detector, which generates a direct electrical readout and therefore enables the measuring of the angle of incidence of up to 60° or the linear polarization state of light, in a spectral region, which is determined by the active material. Our results could furthermore lead to novel organic Bragg SPP-based sensor for a number of applications.

## 1. Introduction

In recent years, substantial research in the field of nanophotonics has targeted the understanding and manipulation of light–matter interactions on the nanoscale. In particular, metallic nanostructures that allow the excitation of plasmons, such as localized surface plasmons (LSPs) on isolated metal nanostructures, surface plasmon polaritons (SPPs) propagating at metal–dielectric interfaces or gap plasmon polaritons (GPPs) on metal–insulator–metal interfaces and the coupling of plasmonic modes gained great attention and can be addressed and utilized [1,2,3]. Consequently, the generation or filtering of colors [4,5], the spatial redirection of light [6,7,8], extraordinary optical transmission (EOT) [9,10], as well as optical cloaking and light trapping in optoelectronic devices [11,12,13,14] have been demonstrated over the last years. Nanogratings have attracted much attention due to easy excitation of Bragg SPP plasmons by diffraction and direct coupling of the wave vector of the incoming light to the reciprocal lattice vector of a grating [1,15,16,17]. The coupling condition of dispersive SPPs depends on the periodicity of the grating and the angle of incidence and the polarization state of the incoming light [18]. The advantages of Bragg SPPs, such as easy excitation, the angular dependent coupling condition, polarization sensitivity, as well as the field enhancements, are widely used in surface plasmon resonance (SPR) sensors [19,20,21,22], for surface plasmon coupled light emission [23], in photo detectors [11,24] and in plasmonic solar cells [12,25,26,27,28].

Although the angle- and polarization-dependent coupling condition of Bragg plasmon excitation principally allows for the SPP plasmon-based determination of the angle of incidence (AOI) or the polarization state of light rays, nowadays, non-SPP-based devices are used to determine the AOI. Such devices often consist of bulky and complex optical systems with lenses, apertures, and sensor arrays, e.g., CCD-cameras, light field cameras, or sun sensors. Light field cameras based on the Talbot effect [29,30,31] simultaneously capture information on intensity and direction of the light in the far field by angle dependent intensity patterns on a photodetector generated by micro lenses or a diffraction grating [32,33,34,35]. Sun sensors often use pinholes or apertures placed in a distance in front of an active pixel array to determine the position of the sun, stars, or the location coordinates of space-crafts [36], satellites, or Mars rovers [37]. In contrast, polarization sensitive photoreceptors are used for navigation and water detection by insects in nature. For instance, desert ants (genus *Catraglyphys*) use celestial polarization patterns and a high level of processing to find direct routes into their home nest [38].

Here, we follow the examples in nature and propose the idea of an organic Bragg SPP-based device to enable angle- and polarization-sensitive detection. We investigate the combination of a flat, thin film organic solar cell with integrated periodic metallic nanostructures. Although operation of such sensors in the visible spectral range are highly desirable, their realization is challenging, and the number of studies on this specific topic is rather moderate. There are a few articles reporting on polarization and wavelength-selective photodetectors based on SPP enhanced photoconductivity in tunnel junctions [24,39,40]. For a fixed angle and a fixed wavelength, Turker et al. [41], and very recently Saito et al. [20] and Tsukagoshi et al. [42], reported on SPR sensors based on a metal grating coupler embedded onto a diode, capable of detecting changes in the refractive index of the surrounding medium without the need of an external readout setup. Senanayake et al. [11] fabricated a surface plasmon enhanced photodetector based on nanopillars, which exhibits an angle dependent photo-response due to excitation of SPPs in the infrared spectral region. In addition, studies on plasmonic solar cells with incorporated periodic metallic nanogratings need to be considered as well. However, in only a few of the studies, the angular response due to SPP coupling was correlated with the performance of plasmonic optoelectronic devices based on nanogrids [12,13,43,44,45,46,47,48,49,50]. In most studies, the devices either lack of clear plasmonic modes in the wavelength region where charge carriers are generated, or the plasmonic influence on the device performance is not significant. Moreover, most studies do not provide angle dependent measurements or discuss the discrepancies between optical and electrical measurements in detail.

In this article, we report the design and fabrication of an angle and polarization sensitive plasmonic detector based on a nanostructured thin film organic solar cell. The nanostructured semi-transparent metallic bottom and top electrodes of the cell allow for light coupling to Bragg plasmons. For both sides of incidence (bottom or top illumination), we find a unique response as a function of the AOI and the linear polarization state of light. Our findings could lead to new types of SPP-based sensors for various applications. One possible application could be the detection of the AOI similar to a sun sensor, in the visible or IR spectral region. Our device is able to detect incoming light for large AOI of at least 60° in a small and adjustable spectral range. Additionally, the design could be used as integrated organic SPR sensors, which allow direct integration into microfluidic or lab-on-a-chip devices and would enable a direct electrical readout of the signals without the need for sophisticated external readout hardware.

## 2. Materials and Methods

### 2.1. Materials

OrmoStamp was purchased from micro resist technology (Berlin, Germany). Poly(3,4-ethylenedioxythiophene):poly(styrene sulfonate) (PEDOT:PSS) Clevios P.Al 4083 was purchased from Heraeus (Hanau, Germany), diluted with water, and 0.7% Zonyl as surfactant was added. Zonyl was purchased from Sigma-Aldrich (Vienna, Austria). The poly(3-hexylthiophene-2,5-diyl) (P3HT) was purchased from Rieke metals (Lincoln, NE, USA) and the [6,6]-Phenyl C61 butyric acid methyl ester [60] PCBM was purchased from Solenne BV (Groningen, Netherlands). P3HT and PCBM were mixed in a ratio of 1:0.7, dissolved in chlorobenzene, and steered for 1 day at 70 °C. To determine the layer thicknesses, each layer was individually spin coated (4000 rpm) on a silicon substrate (as delivered) and the thickness was measured on a scratch made with an scalpel by atomic force microscope (AFM, Bruker corporation, Billerica, MA, USA). The thicknesses of the PEDOT:PSS and P3HT/PCBM layers were 15 and 86 nm, respectively.

### 2.2. Device Fabrication

**Figure 1:**
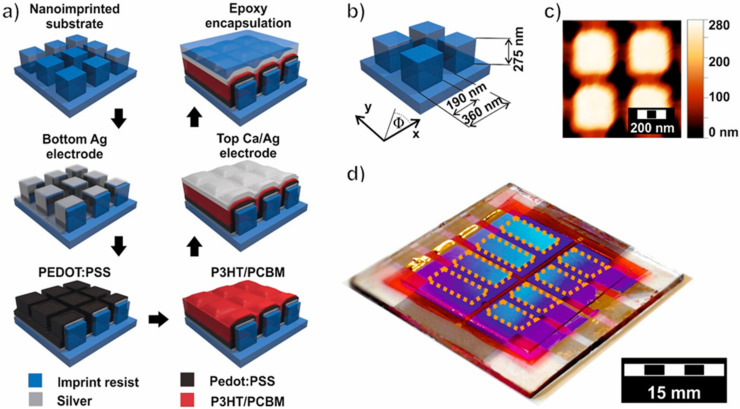
Manufacturing and detector details. (**a**) Fabrication of nanostructures on a glass substrate by nanoimprint lithography, followed by Ag deposition of the bottom electrode. Spin coating of PEDOT:PSS and P3HT/PCBM, metal deposition of the top electrode and encapsulation. (**b**) Schematic of a unit cell. (**c**) AFM topography image of the nanoimprinted substrate. (**d**) Photograph of the final device in true colors, comprising eight individual cells confined by the spatial overlap of the electrodes (indicated by dashed orange lines).

The fabrication steps are schematically depicted in Figure 1a. Firstly, a 2D square lattice of nanostructures is imprinted on a 25 × 25 mm^2^ glass substrate by UV-based nanoimprint lithography (UV-NIL) as described elsewhere [51]. OrmoStamp [52] was used as UV-curable nanoimprint resist. The nanostructures were selected to exhibit the (+1, 0) Bragg SPP mode within the spectral region of λ = 350–650 nm, overlapping with the absorption of the P3HT/PCBM. For this purpose, a silicone NIL master with nanostructures, written with e-beam lithography and successively etched in the silicone, was used. The nanostructures have a feature size of 190 nm, in x and y, and a height of h = 275 nm, as sketched in Figure 1b. The unit cell size of the square lattice is P_x_ = P_y_ = 360 nm. Figure 1c shows a typical topography of the nanoimprinted substrate measured with an AFM. In Figure S1, additional AFM measurements and an electron microscopy image of the cross section through the detector are presented. The bottom electrode is a semi-transparent 25 nm thin silver (Ag) layer deposited by thermal evaporation directly onto the imprinted substrate resulting in a nanomesh that contains square holes at the bottom and square layers on top of the 275 nm high posts. Subsequently, thin PEDOT:PSS hole-conduction layer was spin coated directly on top of this nanostructured electrode and annealed for 30 min at 150 °C on a hotplate (adapted form [53]). This was followed by a thin photoactive layer (P3HT/PCBM), which was spin coated and annealed for 5 min at 80 °C on a hotplate. The nominal thickness of the PEDOT:PSS (14 nm) and P3HT/PCBM (86 nm) layer was determined by spin coating the corresponding layers on silicon substrates. On the nanostructured substrates, the spin coated active layer generates a wavy surface with a periodicity given by the underlying NIL pattern. After coating of the active layer, the topography height of around 140 nm was measured (Figure S1d). Unless mentioned otherwise, the semi-transparent top electrode consists of a 10-nm calcium (Ca) and 50-nm Ag layer deposited, in a nitrogen atmosphere in a glove box, on top of the spin-coated polymers, which gives a solid corrugated metal electrode with a topography height of about 140 nm (Figure S1). The device was encapsulated with UV-curing epoxy resin and a 130-µm-thick glass cover slide in the glove box, in order to prevent degradation. A photograph of a final device comprising 8 cells is shown in real colors in Figure 1d. The individual cells are defined by the spatial overlap of the bottom electrode and the individual top electrodes (indicated by dashed orange lines).

### 2.3. Optical Characterization

Angular-resolved zero-order transmission (T) and zero-order reflection (R) measurements were performed using an in-house built setup. The samples were illuminated by a broadband white light source (tungsten halogen, UV-IR) coupled to a fiber and slightly focused to a round spot with a diameter of 1 mm. T/R measurements were performed using linearly polarized light (GT 10-C Glan-Taylor Calcite Polarizer, Thorlabs, Bergkirchen, Germany) with the electric field either perpendicular (s-polarized) or parallel (p-polarized) to the plane of incidence. Polarization was controlled by turning the polarizer. All optical measurements and electrical characterization were carried out by setting the incidence plane to Φ = 0° azimuthal angle with respect to the nanostructure orientation. The measurements were performed either with incidence through the nanostructured bottom electrode (bottom electrode incidence spectra, BI) or through the semi-transparent corrugated top electrode (top electrode incidence spectra, TI). The transmitted or reflected light was collected by a lens, collimated into an optical fiber and detected with a CCD spectrometer (B&W Tek, BRX112E-V, Newak, DE, USA, range 400–1050 nm). The angle of incidence θ was varied in 2° steps for transmission measurements, which was a good tradeoff between resolution and measuring time. For each angle increment, 25 spectra were accumulated for 40-ms exposure time each. The resulting angle dependent spectra were then corrected by the response function of the setup to yield the transmittance. Reflection measurements were performed using the same setup in 5° increments. Each spectrum was accumulated 25 times with 10-ms exposure time. The resulting angle dependent spectra were again corrected by the response function of the setup to yield the reflectance.

### 2.4. Electrical Characterization

I–V characteristics of the devices were measured on an Oriel solar simulator using an AM1.5 spectrum. The external quantum efficiency (EQE) was determined using a setup consisting of a Xenon lamp, a monochromator, a linear-polarizer, a chopper wheel, and a lock-in amplifier (Stanford Research System SRS830, Sunnyvale, CA, USA). The samples were mounted on a manual rotation stage with the cell under investigation centered on the rotation axes illuminated with a monochromatic light spot of rectangular shape of 2 × 0.5 mm^2^. For determining the light spectrum and the number of incoming photons, a calibrated photodetector (Hamamatsu S2281-01, Herrsching am Ammersee, Germany) was used. Angle dependent EQE measurements were performed by rotating the sample in 5° steps from 0° to 60° incident angle with 0° being the normal incidence. For each incremental step, the wavelength of the monochromatic excitation light was scanned at λ = 305–700 nm in 6-nm steps. All cells were checked for degradation effects before and after performing EQE measurements. No cell degradation was observed during the measurements and all cells showed a homogenous response over the complete active area.

### 2.5. Numerical Simulation

All simulations were performed in the frequency domain with the commercial finite element solver COMSOL™ (Burlington, MA, USA), version 5.2, using the Wave Optics package. The geometrical parameters are taken from scanning electron microscope (SEM, Zeiss1540 XB Oberkochen, Germany) and AFM measurements as described in Section 2.2. The refractive indices of the epoxy and the nanoimprinted structure were assumed to be purely real with a value of *n* = 1.5. The dielectric functions of silver and P3HT were taken from Johnson and Christy [54] and Ng et al. [55], respectively.

## 3. Results

Our proposed angle dependent detector consists of a 2D square lattice nanoimprinted on a substrate and a solution processed thin film organic solar cell on top (Figure 1). The nanostructures were selected to have Bragg SPP modes in the absorbing wavelength region of P3HT/PCBM. The top and bottom electrodes exhibit different optical properties as a consequence of their different morphologies. As stated above, the top electrode is a continuous corrugated layer with structure heights of about 140 nm, while the bottom electrode is a nanomesh that contains square holes at the bottom and square layers on top of the 275-nm high posts. Compared to an open hole array, such a blocked hole array can transmit more light, due to coupling of SPPs and LSPs, known as extraordinary optical transmission (EOT) through blocked holes [10].

To investigate the plasmonic effect of the two different electrodes on the device performance, we carried out optical and electrical characterizations of the sample as a function of the angle of incidence (AOI, θ) for both sides of illumination, top incidence (TI) and bottom incidence (BI). The optical characterization reveals the plasmon-coupling conditions, whereas the electrical characterization, especially the external quantum efficiency (EQE) measurements in the wavelength region between 350 and 650 nm, unveil the direct plasmonic influence on the charge carrier generation in P3HT/PCBM. Additionally, I–V measurements were performed on full devices in order to characterize the performance and the diode behavior.

**Figure 2:**
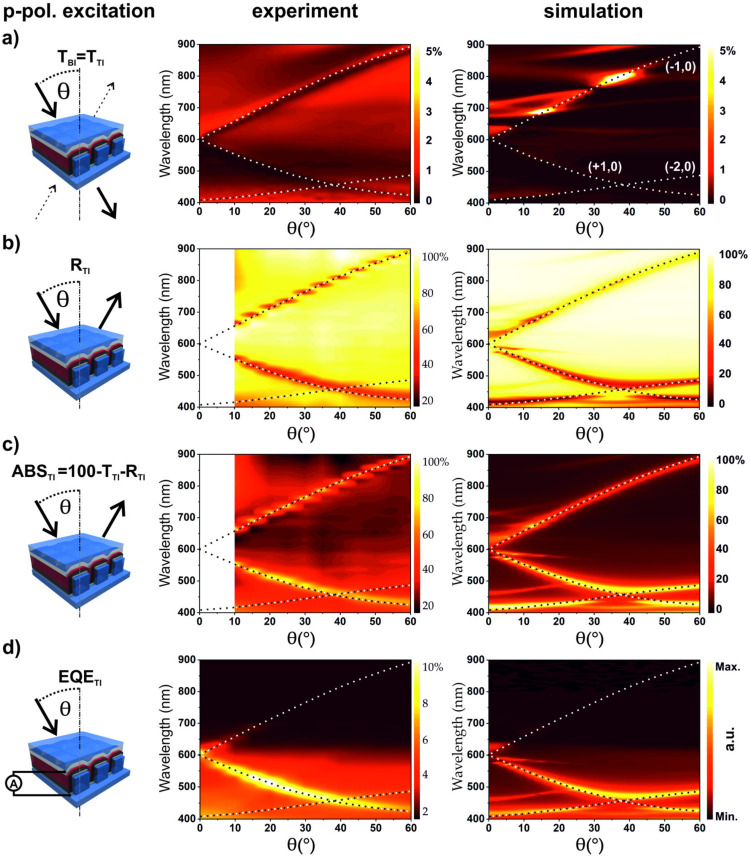
Schematics (left panel) for Angle-resolved device characterization for p-polarized excitation, incident on the top electrode (TI stands for top incidence). Middle panels show experimental data and right panels show corresponding simulated spectra. (**a**) Transmission (T) spectra; (**b**) reflection (R) spectra; (**c**) absorption calculated from T and R; and (**d**) external quantum efficiency (EQE) characterization. The right panel in (d) shows a simulation of the energy absorbed in the active layer. The abrupt decrease in EQE between 600 and 650 nm is caused by the absorption edge of the active material. The incidence plane was set to Φ = 0° azimuthal angle for all measurements. Dispersive SPP modes are clearly visible in all graphs, and calculated SPP modes are shown by dashed lines.

Figure 2 shows the experimental device characterization for excitation with light incident through the top electrode (TI) in the case of p-polarization. The left panels sketch the respective experimental situation, the middle panels show the experimental results, and the right panels show the numerically simulated spectra as a function of wavelength and angle of incidence. The simulations were carried out with COMSOL Multiphysics^®^ (cf. Section 2.5 for more details). It should be noted that the simulation results strongly depend on the properties of the materials. There is an especially big disagreement in the literature on the complex refractive index (RI) of the active material P3HT/PCBM. Figure S2 shows real and imaginary parts of P3HT/PCBM refractive index taken from three different sources [55,56,57]. Despite significant variations of the RI data, our simulation fits qualitatively well to the obtained experimental results in case of the RI data from Ng et al. [55], which we use for all simulations shown in the main text. The Bragg SPP coupling condition allows analytically calculating the Bragg SPP modes dispersion (cf. the Supplementary Materials). The white and black dashed lines in the panels represent the (±1, 0) and (+2, 0) dispersive modes of the SPP at the silver/epoxy interface. They correspond well with the main measured and simulated dispersive features; however, some additional dispersive and non-dispersive features are visible in both simulations and experiments.

The total transmission through the device (Figure 2a) is below 2%, with clearly visible Bragg SPPs in the spectrum. In Figure 2b, the reflection measurements reveal that the metal top electrode acts as a mirror with a reflectivity of up to 80%. Only for light fulfilling the SPP coupling condition, the reflection drops down to 20%. Additional insights can be gained by the absorption A = 100%-T-R, as shown in Figure 2c. The experimental absorption maxima are consistent with the excitation of dispersive (±1, 0) SPP modes. Light, fulfilling the coupling conditions to the SPP, is absorbed with high efficiency within the device. Particularly, we want to address the maximum in absorption at shorter wavelengths (below 610 nm), which originates from coupling to the (+1, 0) mode. For larger AOI, anti-crossing with the (−2, 0) mode is apparent in the simulation, which is absent in the experiment. In general, energy coupled to SPP modes could be dissipated in the metal as Ohmic losses, be reemitted, or it could get absorbed in the dielectric or in the photoactive layer. Energy absorbed in the photoactive layer creates excitons, which can decay in charge carriers or recombine radiatively or non-radiatively. The external quantum efficiency (EQE) should represent a convolution of the absorption spectrum of the active layer and the plasmonic modes of the device. This can be nicely observed in Figure 2d (middle), where the measured EQE is plotted as a function of the incident wavelength and AOI. Due to the band edge and low absorption in the P3HT/PCBM above 650 nm, the (−1, 0) SPP mode is very weak in the EQE measurement, but measurable up to around 700 nm. In contrast, the (+1, 0) SPP mode is well resolved in the EQE measurement. The measured EQE at λ = 515 nm for off-mode (θ = 0°) and on-mode (θ = 20°) condition are EQE_TI_ = 3.02% and EQE_TI_ = 10.9%, respectively. This gives a relative enhancement factor of 3.6, which leads to a strong angular dependent signal for p-polarized light. At λ = 686 nm, at the band edge of P3HT/PCBM, the EQE_TI_ = 0.103% for the off-mode case and for the on-mode EQE_TI_ = 0.539%. This results in a SPP-induced enhancement of 5.2 at the band edge. One can indirectly access the EQE by simulating the absorbed energy just in the active layer (Figure 2d, right). The dispersion of the absorbed energy in the active layer is in excellent agreement with the dispersion of the experimentally measured EQE (Figure 2d, middle). Both the (+1, 0) mode and the onset of the (−1, 0) mode are well resolved. The difference is that, in the simulations more modes are prominent, the anti-crossing of the (+1, 0) mode with the (−2, 0) mode is more pronounced, and the (−2, 0) mode is clearly visible in the simulation compared to the experiments.

**Figure 3:**
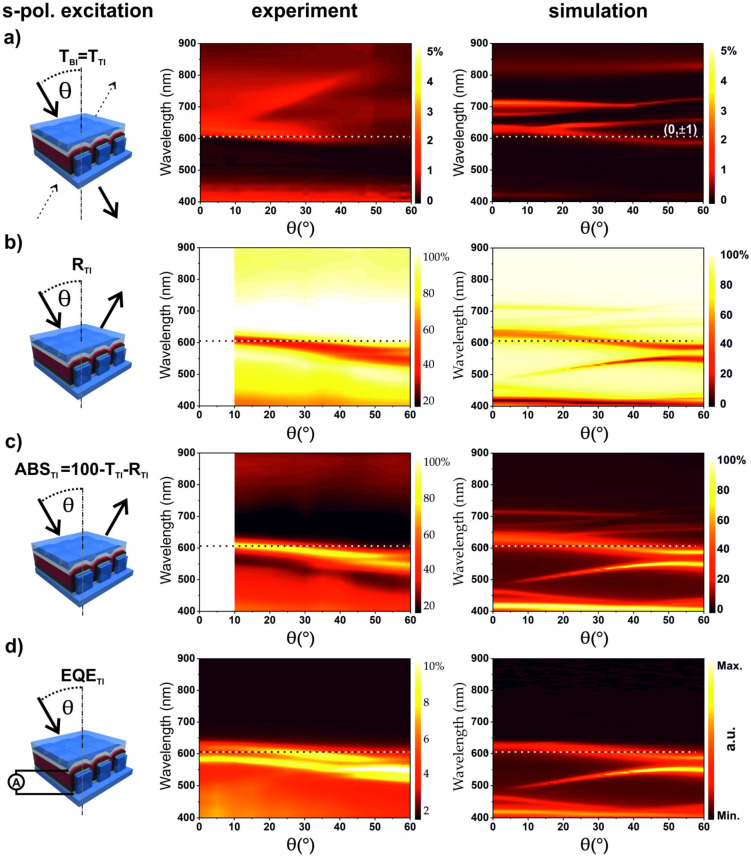
Schematics (left) for angle-resolved device characterization for s-polarized excitation with top incidence (TI) (middle) and corresponding simulated spectra (right). (**a**) Transmission (T) spectra; (**b**) reflection (R) spectra; (**c**) absorption measurement derived from T and R; and (**d**) external quantum efficiency (EQE) characterization. The simulation in (**d**) shows the absorbed energy in the active layer. The plateau at 600–650 nm is caused by the absorption edge of the active material overlapping with the (0, ±1) SPP mode. The incidence plane was set to Φ = 0° azimuthal angle for all measurements. SPP modes are clearly visible in all graphs, and calculated SPP modes are shown by dashed lines.

The optical and electrical characterizations in case of s-polarized light are shown in Figure 3. One notes that all optical and electrical characteristics at normal incidence are identical to the corresponding characteristics for p-polarization, as one expects from the symmetry of the nanostructures. The experimental and simulated transmission for the device is shown in Figure 3a. The measured transmission for s-polarization is again below 2% and shows an almost angle independent absorption edge at around 610 nm and a dispersive feature with higher transmission starting at 660 nm for θ= 15° until 790 nm for θ = 40°. The drastic change in transmission at λ = 610 nm, is caused by the change due to the absorption edge of P3HT/PCBM (see Figures S2 and S4f) and an underlying SPP mode. Reflection and absorption spectra (Figure 3b,c) show in detail the (±1, 0) SPP mode, which is only slightly dispersive in the case of s-polarization, in contrast to p-polarization. In comparison to the absorption (Figure 3c, middle), the EQE measurement (Figure 3d, middle) shows two distinct modes in the wavelength region between 550 and 650 nm. The appearance of two modes in the EQE measurement instead of one is most likely caused by a slight rotation of the sample during the measurement leading to a mode splitting.

The corresponding simulations are presented in Figure 3a–d (right). Clear Bragg SPP modes are also present in the simulation results and agree well with the measurements. However, again, the simulation results show more observable SPP modes as well as their evolution for all calculated spectra. The main (0, ±1) mode is prominent in the simulation as well as in all measurements. Two essential features of the optical properties should be highlighted here: First, for oblique incidence, a clear polarization dependent behavior is visible when comparing the EQE for p-polarization and s-polarization, (Figure 2d and Figure 3d, respectively). Second, SPP modes excited with the s-polarization exhibit very limited dispersion in contrast to SPP modes excited with p-polarization.

**Figure 4:**
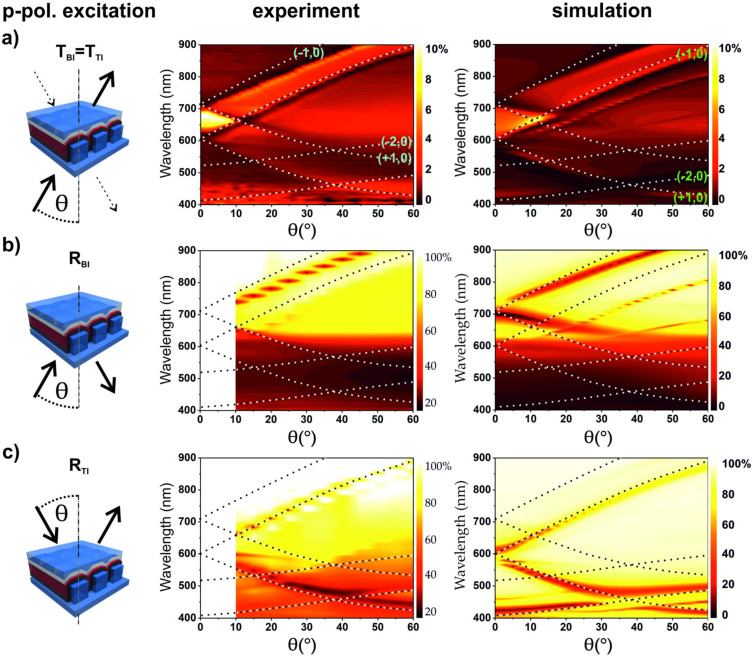
Schamatics (left) for side and angle resolved device characterization with p-polarized excitation (middle) and simulated spectra (right) of a half-cell device without a bottom electrode. (**a**–**c**) Zero-order transmission and side dependent reflection measurements. The incidence plane was set to Φ = 0° azimuthal angle for all measurements. Side dependent reflection measurement shows clear differences in SPP mode excitation. Dashed lines show the analytically calculated Bragg SPP modes at metal–epoxy (green labels in the simulation results) and metal–P3HT/PCBM interfaces (light blue labels in measurements).

To determine the origin and location of the observed SPPs and to exclude the influences between both metal electrodes, half-cell devices were fabricated, optically characterized, and numerically simulated. A half-cell device with top electrodes only (Figure 4) was fabricated to investigate SPP modes at the metal–P3HT/PCBM and at the metal–epoxy interfaces. Additionally, devices without electrodes i.e., patterned polymer layers, were fabricated in order to get information on polymer absorption (see Figure S4).

Figure 4 shows the side dependent optical characterization of a half-cell device without a bottom electrode for p-polarized excitation together with analytically calculated SPP modes. The modes on the P3HT/PCBM–Ag interface are labeled in the experimental results of Figure 4a, and the modes at the metal–epoxy interface are labeled in the simulation results. The transmission measurements as well as the simulated spectra presented in Figure 4a reveal SPP modes at both metal–dielectric interfaces. The simulations disclose that the (±1, 0) SPP mode is located at the Ag–epoxy interface, starting at around λ = 606 nm for normal incidence (θ = 0°), while another (±1, 0) SPP mode at the P3HT/PCBM–Ag interface starts at around λ = 707 nm for normal incidence (θ = 0°). The energetic (wavelength) difference between those SPPs arises from the refractive index differences between the P3HT/PCBM, with average real part of the RI in the visible spectral range around 1.9, and the epoxy with a purely real refractive index of *n* = 1.5. The excitation of the SPPs at the respective interfaces is confirmed additionally in the reflection measurements presented in Figure 4b,c. In the case of BI excitation, the reflection spectra show primarily the SPP modes at the P3HT/PCBM–metal interface, while TI excitation predominantly couples to SPP modes at the metal–epoxy interface.

This shows that SPPs at both interfaces at the top electrode are present in the case of the half-cell device and are clearly visible in the experiments and the simulations. This is in contrast to observations from the full-cell device (Figure 2 and Figure 3), where only the SPP modes at the metal–epoxy interface are prominent and visible, as revealed by the transmission measurements in Figure 2a. The absence of the P3HT/PCBM–metal SPP modes in the full device suggests that the bottom electrode strongly affects the Bragg SPP coupling conditions. These modes are either suppressed due to the presence of the bottom electrode or energetically shifted due to plasmonic coupling between the modes at the top and the bottom electrodes. Specifically, the part of the bottom electrode on top of the nanoimprinted pillars, which is very close to the top electrode, could affect the coupling.

**Figure 5:**
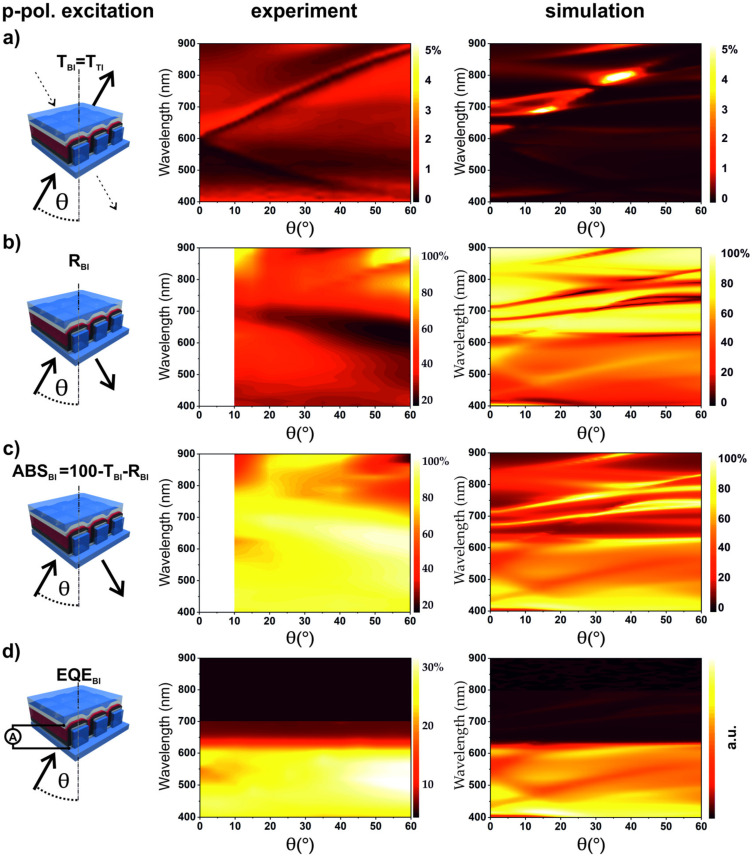
Schematics (left) for angle resolved device characterization with p-polarized excitation for bottom incidence (BI) (middle) and simulated spectra (right). (**a**) Transmission (T) spectra; (**b**) reflection (R) spectra; (**c**) absorption measurement derived from T and R; and (**d**) measured external quantum efficiency (EQE) characterization and the simulated absorbed energy in the active layer. The abrupt decrease in EQE between 600 and 650 nm is caused by the absorption edge of the active material. The incidence plane was set to Φ = 0° azimuthal angle for all measurements.

Next, the influence of the bottom electrode on device characteristics is investigated by angle-resolved measurements. In Figure 5, the angle resolved device characterization and simulated spectra for p-polarized excitation are shown. The transmittance for bottom incidence is the same as for top incidence within experimental error, as presented in Figure S5. Absorption, ABS_BI_, and EQE_BI_ are shown in Figure 5c,d, respectively. The reflection, absorption, and EQE for BI differ significantly from the corresponding results for TI. There are no clear Bragg SPP modes visible in the reflection spectra (Figure 5b). Distinct SPP modes are observed neither in the EQE nor the absorption spectra. For BI, the absorption at λ = 350–650 nm is in the range of 80%, which can be attributed to the good absorption of P3HT/PCBM in this wavelength region. The absorption and EQE spectra show a broad spectral minimum, between 500 and 700 nm, for small AOI and a broad maximum for larger AOI in the same region. For the full device, EQE lies between 16% and 29.8% over the whole spectrum and AOI range. By comparing the measured EQE_BI_ with EQE_TI_, one notices that, in the case of TI, the energy coupled to SPP modes is generating less charge carriers. For instance, at 521 nm and 20° AOI, which represents a region with Bragg SPP coupling for TI, the external quantum efficiency for TI is EQE_TI(p-pol)_ = 10.1%, while for BI, the EQE_BI(p-pol)_ = 21.9%. Furthermore, the absorptions at 521 nm and 20° AOI for TI and BI are comparable, ABS_TI(p-pol)_ = 66.34% and ABS_BI(p-pol)_ = 71.76%. This confirms that for TI only a part of the energy coupling to SPPs gets absorbed in the active layer, generating excitons and charge carriers. Angle resolved device characterization for BI and s-polarized excitation are presented in Figure S6.

Additionally, our results show that, for bottom incidence, our device acts as an ITO free thin film organic solar cell with no prominent angular dependency. I–V characteristics presented in the Supplementary Materials (Table S1 and Figure S7) show, for BI (TI) through the 25-nm Ag bottom electrode (10-nm Ca/50-nm Ag top electrode), a short circuit current density of J_sc_ = 5.5 mAcm^−2^ (J_sc_ = 1.06 mAcm^−2^), a fill factor FF = 0.62 (FF = 0.56), and an open circuit voltage V_oc_ = 0.6 V (V_oc_ = 0.53 V). The difference in the device characteristics is mainly due to the different transparency of the electrodes. Furthermore, the power conversion efficiency (PCE), under AM1.5 spectrum, is PCE_BI_ = 1.88% when excited through the bottom electrode and PCE_TI_ = 0.4% when excited through the top electrode at normal incidence. The cells show a considerable PCE of up to 1.88% (see Table S1), which is an interesting demonstration for the realization of an ITO-free solar cell using metal electrodes. The efficiency is comparable with other ITO-free P3HT:PCBM organic solar cells with metal electrodes [57,58]. The presented approach might be very interesting due to its simple fabrication without any complex etching or lift-off steps and the ability of upscaling to roll-to-roll NIL processes. However, the maximum EQE of 30% is lower in comparison to optimized ITO–PEDOT:PSS–P3HT/PCBM solar cells reaching up to 60% [59]. Nevertheless, performance optimization was not within the scope of this study. The lower EQE originates from higher reflectivity of the metal bottom electrode compared to an ITO bottom electrode and different work functions.

Since transmission spectra are independent of the side of incidence, they point out that SPPs are excited in either case of incidence. However, for BI, the influence of the SPP modes on EQE is not as prominent. This is very interesting since blocked hole arrays are known to support both SPPs and LSPs [10]. The 25-nm Ag bottom electrode exhibits a number of plasmon modes over the whole spectrum, as shown in the Figure S4 where transmission measurements of the bottom electrode without PEDOT:PSS and P3HT/PCBM layer are presented. However, measurements show that the plasmonic modes of the bottom electrode have no significant influence on the EQE of the final device. For instance, the >50% transmission at normal incidence indicates EOT since transmission through a plain solid 25-nm Ag layer gives a maximum transmission of only around 10% (e.g., λ = 500–900 nm) [60]. Our findings are in good agreement with other reports, as similar designs have been investigated before and often no significant influence due to Bragg modes in the EQE was observed for excitation through the bottom electrode [13,47]. The reason for the difference between BI and TI originates from the different shapes of the electrodes and different reflection spectra. In addition, the bottom electrodes are often made of ITO, and, if metal is used, they are usually very thin to ensure high transmission and therefore SPP modes are not as strong as for the thicker top electrodes.

EQE measurements confirm that only for top incidence, a part of the energy from the Bragg SPP modes excited at the metal–epoxy interface is absorbed in the active layer and generates charge carriers, either by direct absorption of the SPP energy in the active material or by SPP induced high transmission through the top electrode. Although the transmission measurements (Figure 2a and Figure 5a) revealed that the same SPP modes are excited with both TI and BI, the influence on the EQE is only observed for TI. The local electric field distributions in one unit cell, corresponding to BI and TI exaction at 505 nm for various AOI, are displayed in Figure S3. The following two reasons may explain this behavior. First, for TI, the top electrode acts as a very good mirror with reflectivity reaching 80%, whereas, where coupling to SPP at the metal–epoxy interface occurs, reflection drops to 20%. The EQE measurements show that only incident light at the SPP wavelength is efficiently absorbed by the active layer and generates charge carriers. Secondly, SPPs at the top metal–epoxy interface are not excited efficiently in the case of BI, and, thus, there is no significant influence on the EQE. The reason, therefore, might be that up to 96% of the light gets absorbed in a single pass through the active layer (see Figure S4) by leading to only few percent of the incoming light reaching the metal top electrode. Indeed, this light could excite plasmons at the P3HT/PCBM–metal interface, but in contrast to TI these SPPs should lower the respected EQE. However, measurements point out that these SPPs are either not present or simply too weak to significantly contribute to a change of the EQE.

Our device architecture facilitates angle and polarization dependent response when using TI, allowing for the conceptual design of a Bragg SPP-based organic detector, sensitive to both, the AOI and the polarization state of light. The most important features enabling an angle and polarization dependent response are the corrugated semi-transparent top electrode supporting Bragg SPP modes, an active material in close contact with the nanostructured electrode providing a spatial overlap of the electromagnetic modes with the charge generation regions, and the spectral overlap of the SPP modes with the absorption of the active material which facilitates the charge generation. Light coupled to SPPs on the metal–epoxy interface and subsequently absorbed in the active material creates a photocurrent, which can be used as detector signal and is strongly linked to the coupling condition for SPPs, as can be seen in Figure 2d and Figure 3d.

The SPP coupling condition depends on the epoxy encapsulation, the photoactive layer, the electrode materials, and the unit cell size (see Supplementary Materials). The use of epoxy encapsulation with a different refractive index could shift the coupling conditions, which allows a shift of the resonances. The active layer itself should not drastically influence SPP coupling condition, since SPPs are located at the metal–epoxy interface, but a change of the active material would change the response dramatically due to the change in the absorbing and thus charge generating spectral region. The active material P3HT/PCBM absorbs light up to 650 nm, which gives a negative angular response, since the resonance of the (+1, 0) mode blue-shifts for increasing AOI. In comparison, a detector using another active material, absorbing for example at λ = 650–900 nm, would exhibit a positive angular response due to the redshift of (−1, 0) SPP mode for increasing AOI. Our findings further show that the choice of the top electrode material and thickness strongly influences the signal strength (Figure S8). A thin top electrode shows higher intrinsic transmission but a lower signal to background ratio compared to thicker ones. The 10-nm Ca/50-nm Ag top electrode used in this work represents a good tradeoff. Another influence on the coupling condition of SPPs arises from the unit cell size of the corrugated top electrode. Smaller unit cells would blueshift the resonances, whereas larger unit cell sizes would redshift the resonances (Figure S8b). Furthermore, a rectangular unit cell or line and space pattern [39], instead of a square unit cell, would change SPP resonances for p- and s-polarization and hence could improve the polarization sensitivity.

**Figure 6:**
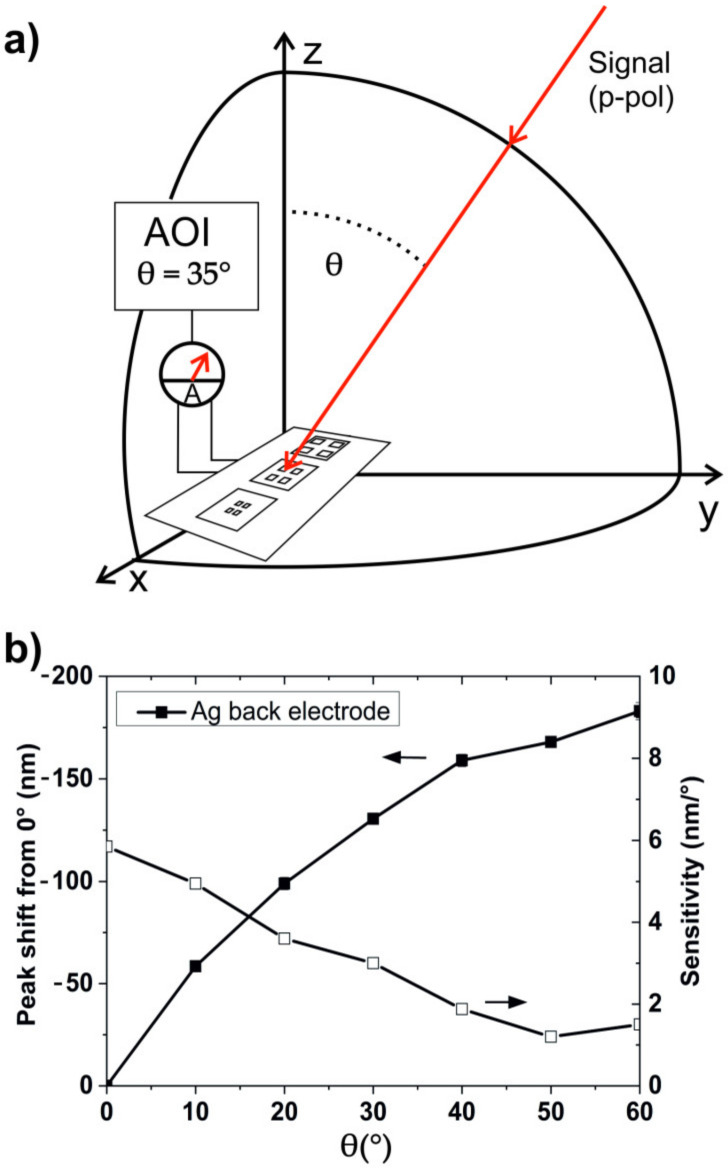
(**a**) Schematic illustration of the detector concept used for the detection of the AOI of incoming light. (**b**) Sensitivity and spectral shift of the resonance peak depending on the angle of incidence.

This effect could be utilized to detect the AOI by using a narrow band excitation. The generated response strongly depends on the AOI and the polarization state of the light. It should be noted here that it is not possible to determine both azimuth and polar angle simultaneously. Therefore, for the proposed application as AOI detector, the p-polarization between emitter and detector must be guaranteed. The concept of a detector device, capable of detecting the AOI of an incoming light ray, is illustrated in Figure 6a. The proposed device would comprise a detector array of a number of individual detectors. Additionally, a linearly polarized light source with a narrow emitter wavelength is necessary. The polarization of the emitter needs to be set to p-polarization. The individual detectors would have different responses in order to be able to calculate the properties of the incoming light. The necessary different responses could either be created by using different epoxy encapsulations, different unit cell dimension of the nanostructures, or different absorber layers for the individual detectors. For example, by using nanoimprint lithography for the substrate fabrication, one could fabricate all nanostructures, in one imprint step, by imprinting a number of different detectors with different unit cell sizes. All other process steps would stay the same.

The angular sensitivity of the detector can be measured by the spectral shift of the Bragg plasmon resonance while changing the AOI by 1°. Our detector shows an angular sensitivity of up to 6 nm for p-polarized light, as can be seen in Figure 6b. The calculated SPP modes for different unit cell sizes are shown in Figure S8. We propose to take advantage of the characteristics of the (−1, 0) mode, namely the dispersion over a spectral range of 180 nm. An array of 12 individual pixels (detectors) is necessary for the detection of a signal covering an AOI from 0° to 60°, by using a signal with a narrow spectral width (FWHM) of 15 nm. The benefit of our design is that such a detector can be easily fabricated directly on one substrate in one imprint process by just using a special designed master, containing an arrangement of 12 individual patterns. Additionally, at least one reference cell (with no SPP excitation) for detecting the signal strength might be necessary. A graphical solution for covering the whole AOI range is shown in Figure S9. By using a number of emitter wavelengths, a reduction of individual detectors could be possible. Even using only one detector and a wavelength sweep for the excitation, the determination of the AOI should be possible as long as the detector knows the emitted wavelength at each time. Finally, by comparing the signals of the individual detector, one could calculate the AOI of the signal.

## 4. Conclusions

We successfully designed and fabricated an angle of incidence (AOI) and polarization state sensitive plasmonic device. The device is based on an ITO-free thin film bulk heterojunction organic solar cell, which has two semi-transparent metal electrodes enclosing the PEDOT:PSS hole-conduction layer and photoactive layer P3HT/PCBM. The device was easily fabricated by spin coating and metal deposition on top of a nanoimprinted substrate.

A thorough analysis of the optical and electrical device characteristics, together with simulations, shows a significant influence of the side of excitation, the AOI and the polarization state of the signal on the EQE of the device. With excitation through the thin bottom electrode (BI), the device acts as an ITO-free organic solar cell with a considerable PCE of 1.88% and an EQE of up to 30% without performing any optimization. The most interesting behavior is revealed with top incidence (TI) where the device shows a clear AOI and polarization dependent response. We conclusively present that with TI the coupling of light to Bragg SPP modes on the corrugated top metal–epoxy interface occurs and that this coupling and the subsequent absorption and charge carrier generation in the device allows for an AOI and polarization dependent response. We show that SPP coupling condition can be controlled in several ways, which allows for an adaption of resonance condition, spectral range, and sensitivity. Based on our results for TI, we propose a concept of polarization sensitive Bragg SPP-based detector capable of detecting the AOI of at least up to 60°. In brief, a Bragg SPP-based AOI detector should consist of an array of individual devices showing different responses. This can be utilized by using different unit cell sizes, active materials or epoxy encapsulation materials. The individual SPP-based devices should be arranged in an array to be able to calculate the properties of the angle of the incoming light ray for p-polarized light.

In addition, our detector concept could lead to the realization of novel organic SPP-based sensors for a number of applications. For instance, our sensor concept could be used for an organic integrated SPR biosensor. By exchanging the epoxy with a thin functionalized silica protection layer and an analyte solution, one can detect changes in refractive index instead of AOI. Such a sensor would allow direct integration into microfluidic or lab-on-a-chip devices and would enable a direct electrical readout of the signal without the need of a large external setup. Furthermore, our device fabrication could be a promising strategy to demonstrate angular and polarization dependent behavior when using light emitting materials and operating the sensor as an OLED.

## Supplementary Materials

The following are available online at https://www.mdpi.com/2079-4991/10/9/1866/s1, Figure S1. 3D visualizations of AFM measurements, a sketch as well as a scanning electron microscope (SEM) image of the device in different fabrication stages. Figure S2. Refractive index for P3HT:PCBM from different sources. Figure S3. Electric field distribution in one unit cell for BI and TI for various AOI. Figure S4. Side and angle-resolved characterization on different half-cell devices. Figure S5. Comparison of side dependent zero order transmission measurement for device with 25-nm bottom and 10-nm Ca/25-nm Ag top electrode. Figure S6. Angle-resolved device characterization with bottom incidence (BI) for s-polarized excitation. Table S1. Current–density–voltage characteristics of devices with 25-nm Ag front electrode and different top electrode thicknesses and materials. Figure S7. Current–voltage (J-V measurements) measurements for bottom incidence for a device with 25-nm bottom electrode and 10-nm Ca/50-nm Ag top electrode. Figure S8. Detector response (EQE) for devices with different top electrode thicknesses for normal incidence (TI) for p-polarized light. Figure S9. Necessary number of detectors depending on wavelengths to cover the complete range of AOI.
