# Supplementary material for: Plasmon-Assisted Direction- and Polarization-Sensitive Organic Thin-Film Detector
Source: Nanomaterials (Basel). 2020 Sep 17;10(9):1866. doi: 10.3390/nano10091866 (PMC7559313; doi:10.3390/nano10091866)
Supplement: Supplementary file 1 [file nanomaterials-10-01866-s001.pdf]

nanomaterials

Supporting Information

Plasmon-assisted Direction- and Polarization-Sensitive Organic Thin-Film Detector

Michael J. Haslinger, Dmitry Sivun, Hannes Pöhl, Battulga Munkhbat, Michael Mühlberger, Thomas A. Klar, Markus C. Scharber and Calin Hrelescu

S1. Sample geometrics

Figure S1 is showing 3D visualizations of the topography obtained from atomic force microscope (AFM) measurements (Figure S1a,d), sketches (Figure S1b,e), as well as scanning electron microscope (SEM) images (Figure S1c,f) of the device in different fabrication stages. AFM measurement and sketch of the nanoimprinted substrate (Figure S1a,b). SEM image of the substrate after bottom electrode deposition (Figure S1c). Figure S1d–f are for the sample after the bottom electrode deposition and PEDOT:PSS and P3HT/PCBM spin-coating.

All AFM measurements were made using a Brucker Dimension AFM and SEM measurements are made with a SEM Zeiss 1540XB. The surface topography changed from 275 nm height (Figure S1a) to around 140 nm height after spin coating of PEDOT:PSS and P3HT/PCBM on the imprinted substrate (Figure S1d). It can be assumed that the anisotropic metal deposition of the 10 nm Ca / 50 nm Ag top electrode ontop of the spin coated polymer layer, does not significantly alter the topography of the surface, except adding some minor roughness. This means that the surface geometry presented in (Figure S1d) also represents the surface geometry of the top electrode-epoxy interface.

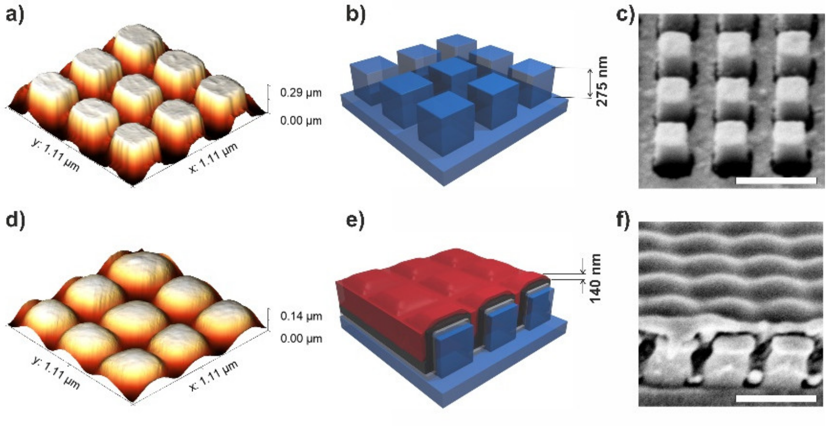

Figure S1. 3D visualizations of AFM measurements, a sketch as well as a scanning electron microscope (SEM) image of the device in different fabrication stages. (**a**,**b**) AFM measurement and sketch of the nanoimprinted substrate. (**c**) SEM image of the substrate after bottom electrode deposition. (**d**–**f**) Characterization of a sample after bottom electrode deposition and PEDOT:PSS and P3HT/PCBM spin-coating. The scale bar is 500 nm.

S2. Calculation of Bragg SPP modes

Bragg SPP modes are two dimensional charge density oscillations, propagating along a metal dielectric interface, which must fulfill the relation, [1]

$$k_{SPP} = k_o \sqrt{\frac{\varepsilon_d \varepsilon_m}{\varepsilon_d + \varepsilon_m}}$$

where ε_m and ε_d are the electric permittivities of the metal and the dielectric. SPPs have a larger in-plane momentum k_{spp} than free space light k_0 due to the discontinuity of the electric permittivity across the metal-dielectric interface. In case of periodic modulation of the metal dielectric interface through nanostructures, Bragg scattering creates an additional momentum compensating the momentum mismatch. The plasmon excitation can be described by [1]

$$\boldsymbol{k}_{||} + i\boldsymbol{G}_x + j\boldsymbol{G}_y = \boldsymbol{k}_{SPP} \;\; and \; \boldsymbol{k}_{||} = |\boldsymbol{k}_0| \sin\theta \cos\phi \;\; \boldsymbol{x} + |\boldsymbol{k}_0| \sin\theta \sin\phi \;\; \boldsymbol{y} \,.$$

i and j are integers specifying the order of the SPP mode and $\boldsymbol{G}_x$ and $\boldsymbol{G}_y$ are the reciprocal lattice vectors of the nanostructures. The in-plane vector $\boldsymbol{k}_{||}$ of the incident wave is a function of the angle of incidence θ and the azimuthal angle Φ [1]. In summary Bragg SPP mode coupling depends on the materials, the unit cell size of the nanostructures, the angle of incidence (AOI), the polarization state, and the wavelength of light.

S3. Refractive index variations found in literature for active material P3HT:PCBM

For COMSOL simulations, the following parameters were used. For the epoxy encapsulation and the imprint material as well as the glass substrate a real refractive index (RI) of n = 1.5 was used. In Figure S2 different RI values from literature are plotted [2–4]. We used the data from Ng. et.al. [3] for our simulations which gave the quantitatively closest results to our experiments.

The dimensions of the pattern were slightly modified in the simulations in order to achieve again simulations quantitatively closer to our experiments. The top electrode thickness was t_{Ag} = 60 nm, t_{Ca} = 5 nm, the periodicity was p = 345 nm and a top electrode curvature of -0.0015*x^2-0.0015*y^2 was assumed.

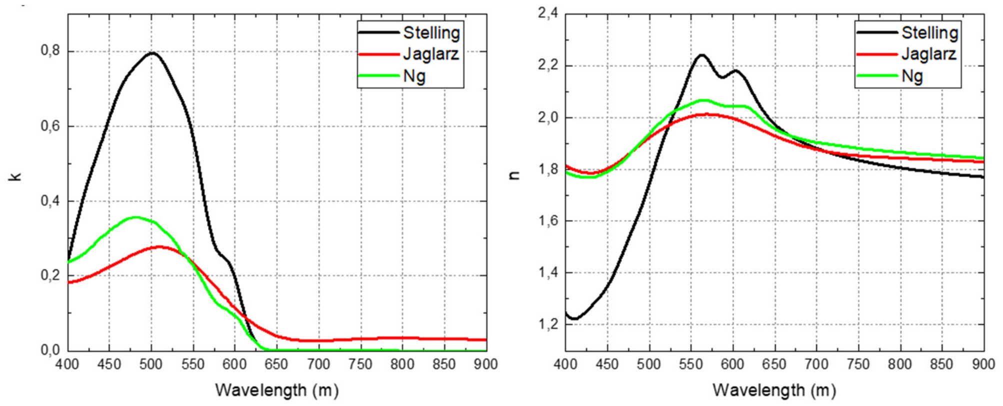

Figure S2. Refractive index for P3HT:PCBM from different sources: Stelling et al. [4], Jaglarz et al. [2] and Ng et al. [3].

S4. Electric field distribution showing one unit cell of the structure for TI and BI for various AOI.

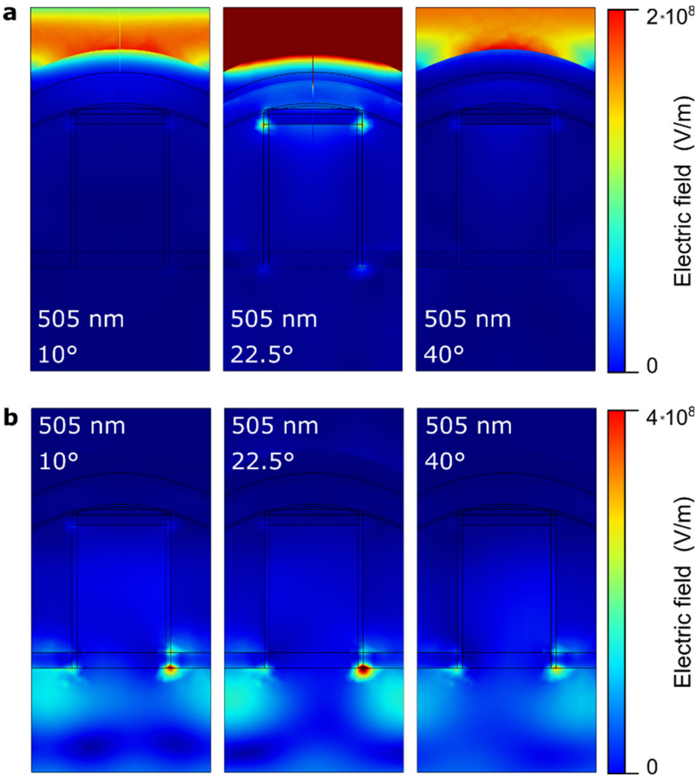

Figure S3. Electric field distribution in one unit cell for: (**a**) (p-pol) top incidence excitation at 505 nm for three AOI. From left to right: 10°, 22.5°, and 40°. At 22.5°, the resonance with the mode leads to the higher field in the active material and hence higher EQE (see Figure 2d). (**b**) (p-pol) Bottom incidence excitation at 505 nm for the three AOI. From left to right: 10°, 22.5°, and 40°. Fields distributions are very similar for all angles of incidence, therefore the field distributions in the active material area are similar and consequently no enhancement in EQE is observed (see Figure 5d).

S5. Side and angle dependent optical characterization on different half-cell devices for p- and s-polarization (10 nm Ca / 50 nm Ag top electrode)

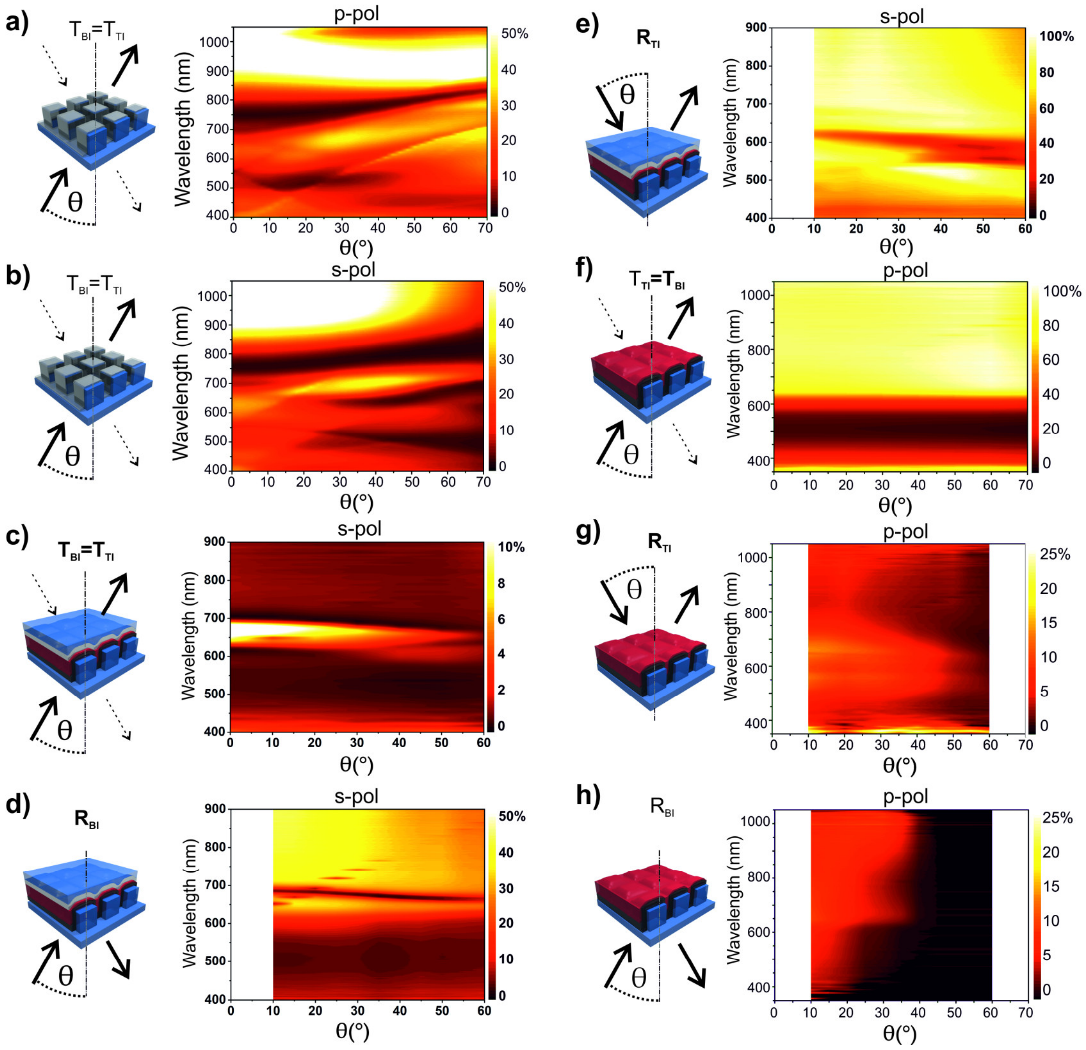

Figure S4. Side and angle-resolved characterization on different half-cell devices. (**a**,**b**) polarization dependent transmission measurement through the device with bottom electrode only. (**c**–**e**) transmission and side dependent reflection measurement for s-polarization for a half cell device without bottom electrode. (**f**–**h**) transmission and side dependent reflection measurement for p-polarization on a half-cell device without bottom and top electrode. For all measurements, the incidence plane is set to Φ = 0° azimuthal angle and p-polarized light was used.

S6. Side dependent transmission measurements

Figure S5 shows side dependent zero order transmission measurements of a device with 25 nm Ag front electrode and a 10 nm Ca / 25 nm Ag top electrode. This device was selected due to the higher intrinsic transmittance compared to a device with 10 nm Ca / 50 nm Ag top electrode presented in the main manuscript. The difference is calculated by $\Delta T = |T_{BI}-T_{TI}|$. The device shows no significant difference in transmission depending the side of excitation, as can be seen in Figure S5d). The small differences < 2.5% in transmittance, are due to the high angular sensitivity of SPP′s and caused by small measurement errors in the angle of incidence. All full devices showed no difference on side of excitation independently of electrode thicknesses.

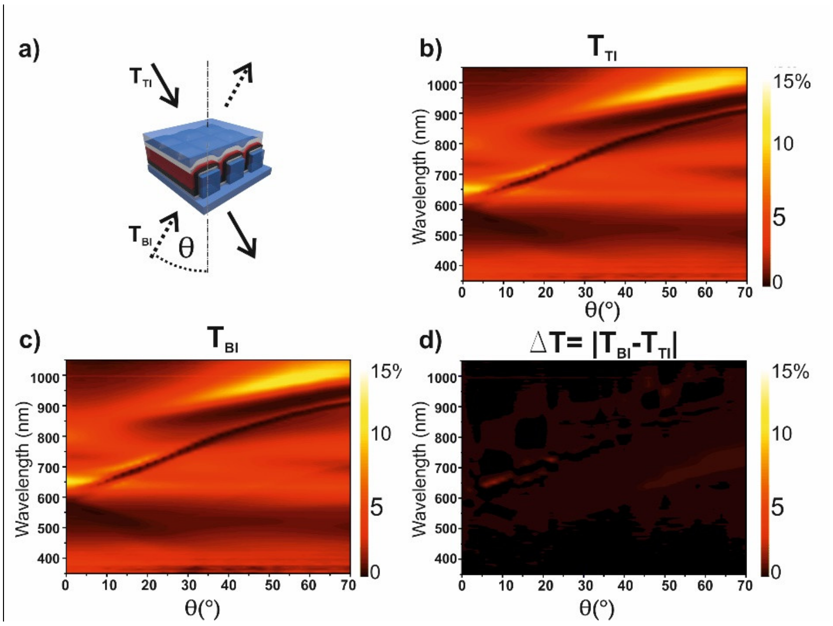

Figure S5. Comparison of side dependent zero order transmission measurement for device with 25 nm bottom and 10 nm Ca / 25 nm Ag top electrode. In (**a**) a schematic illustration of the measurement is shown. (**b**–**d**) Transmission measurement for T_{BI} and T_{TI} together with the difference of both spectra.

S7. Characterization with bottom incidence for s-polarized light (10 nm Ca / 50 nm Ag top electrode)

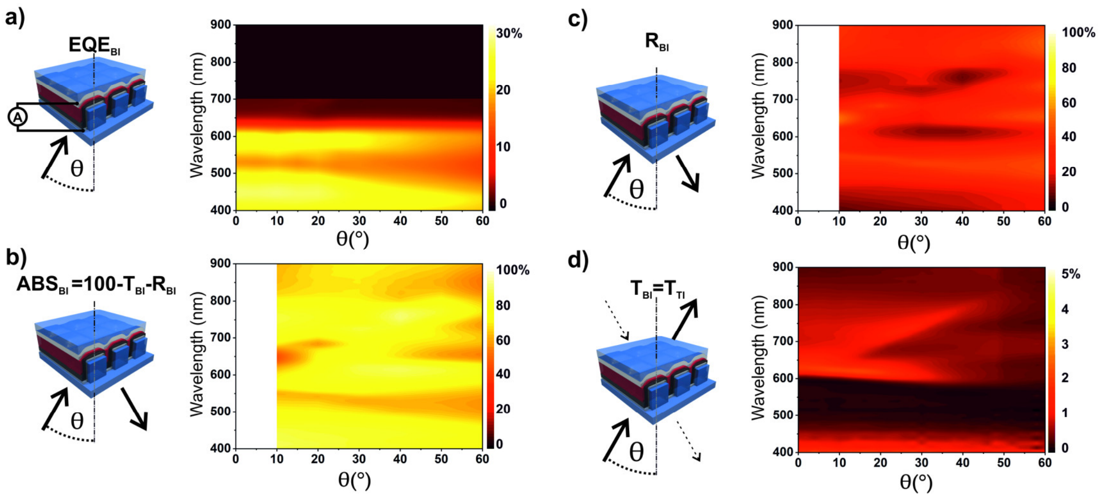

Figure S6. Angle-resolved device characterization with bottom incidence (BI) for s-polarized excitation. (**a**) External quantum efficiency (EQE). (**b**) Absorption derived from the measured T and R. (**c**) Reflection (R) spectra. (**d**) Transmission (T) spectra. The incidence plane was set to Φ = 0° azimuthal angle for all measurements.

S8. Electrical device characterization

This section presents J-V measurements of various devices with 25 nm bottom electrode but different top electrodes. The cells were characterized using an Oriel solar simulator using a AM1.5 spectrum (100 mW/cm²). The results for the side dependent electrical characterization (at normal incidence) and different top electrode thicknesses as well as materials are summarized Table S1. All values are normalized to a detector area of 0.150 cm². The differences for PCE characteristics for BI and TI arise from different thicknesses and therefore different transmission through the electrodes. I-V measurements reveal a high filling factor (FF) of >55% which indicates proper working devices.

The device with 10 nm Ca / 50 nm Ag as top electrode shows side dependent PCEs of PCE_{BI} = 2.578% and PCE_{TI} = 0.400% and a difference of PCE_{BI}/PCE_{TI} = 6.44.

Table S1. Current density voltage characteristics of devices with 25 nm Ag front electrode and different top electrode thicknesses and materials. Measurements made with AM 1.5 G illumination (Oriel solar simulator, 100 mW/cm²), open circuit Voltage (VOC), Shortcut current (JSC), filling factor (FF). All values are normalized to a detector area of 0.150 cm² and averaged over several devices.

Sample orientation / Top electrode	V_{OC} [V]	J_{SC} [mA/cm²]	*FF* [%]	*PCE* [%]	R_s [Ω]
Mode (BI) / 10nm Ca/50nm Ag	0.59 ± 0.004	5.41 ± 0.34	61.3 ± 1.44	1.88 ± 0.16	24.9 ± 4.01
Mode (TI) / 10nm Ca/50nm Ag	0.53 ± 0.002	1.06 ± 0.047	56.2 ± 5.50	0.28 ± 0.02	39.2 ± 10.7
Mode (BI) / 10nm Ca/25nm Ag	0.58 ± 0.002	5.19 ± 0.16	57.2 ± 1.88	1.63 ± 0.13	29.1 ± 4.22
Mode (BI) / 10nm Ca/110nm Ag	0.58 ± 0.002	4.30 ± 0.16	54.8 ± 0.68	1.32 ± 0.06	111.3 ± 13.57

A typical J-V measurement is shown in Figure S7.

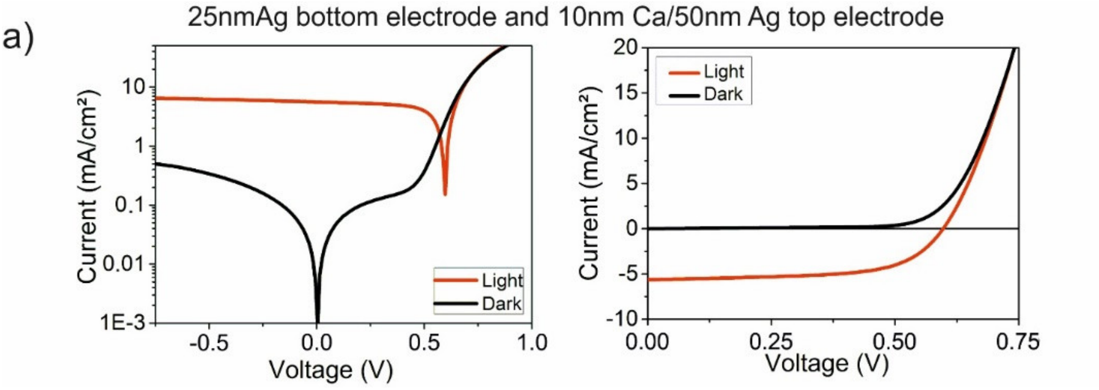

Figure S7. Current-Voltage (J-V measurements) measurements for bottom incidence for a device with 25 nm bottom electrode and 10 nm Ca / 50 nm Ag top electrode.

S9. Angular response of devices with different top electrode thicknesses

Devices with 10 nm Ca / plus 25 nm, 50 nm and 110 nm Ag top electrodes were fabricated and analyzed. The EQEs as a function of the incident wavelength for different excitation angles and thicknesses of top electrodes are shown in Figure S8a. With increasing top electrode thickness, the detector signal, meaning SPP EQE signal, as well as the overall transmitted light decreases. A thin top electrode shows higher intrinsic transmission but a lower on-/off resonance ratio compared to thicker ones. The on-/off resonance ratio is directly visible in Figure S8a and represents the EQE in SPP coupling condition vs the EQE off resonance. For an angle of incidence of 20°, the EQE signal to background ratio (0°) is around 20.63 / 13.06 = 1.6 for the device with 25 nm top electrode. The on-/off resonance ratio for the 50 nm is 10.9 / 3.02 = 3.63 and 0.001 / (4.8×10^{-4}) = 2.105 for the 110 nm Ag top electrode. The device with a 10 nm Ca / 50 nm top electrode as discussed in the main manuscript represents a good tradeoff for a detector.

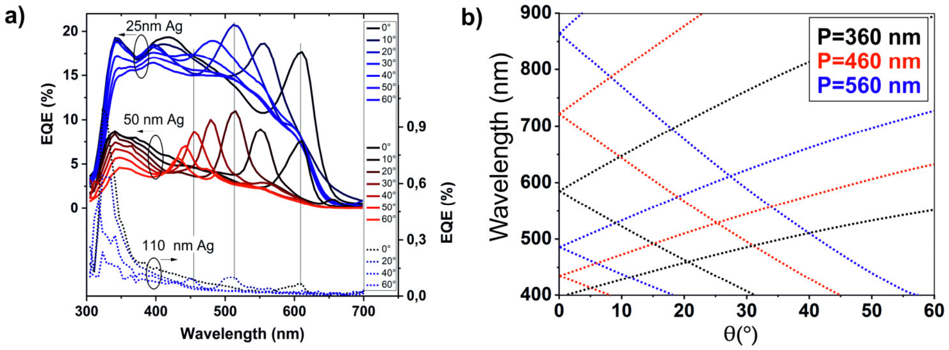

Figure S8. (**a**) Detector response (EQE) for devices with different top electrode thicknesses for normal incidence (TI) for p-polarized light. (**b**) Calculated SPP modes of gratings with different unit cell size.

S10. Graphical solution for the necessary number of detectors for detecting the AOI by using a different number of excitation wavelengths.

A FWHM for the excitation light source of 15 nm was assumed. It was assumed that only (−1, 0) Bragg SPP modes are facilitated by the detector.

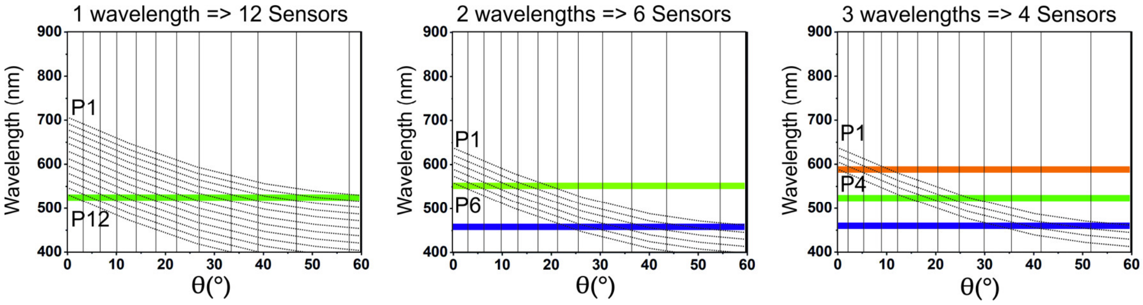

Figure S9. Necessary number of detectors depending on wavelengths to cover the complete range of AOI. P1–P12 represents 12 different detector responses are necessary (right). For two or more excitation wavelengths, the required number of detectors can be reduced (middle and right).

References

[1] H. Gao, W. Zhou, and T. W. Odom, ‘Plasmonic Crystals: A Platform to Catalog Resonances from Ultraviolet to Near-Infrared Wavelengths in a Plasmonic Library’, *Adv. Funct. Mater.*, vol. 20, no. 4, pp. 529–539, Feb. 2010, doi: 10.1002/adfm.200901623.

[2] J. Jaglarz, A. Małek, and J. Sanetra, ‘Thermal Dependence of Optical Parameters of Thin Polythiophene Films Blended with PCBM’, *Polymers*, vol. 10, no. 4, p. 454, 2018.

[3] A. Ng *et al.*, ‘Accurate determination of the index of refraction of polymer blend films by spectroscopic ellipsometry’, *J. Phys. Chem. C*, vol. 114, no. 35, pp. 15094–15101, 2010.

[4] C. Stelling, C. R. Singh, M. Karg, T. A. F. König, M. Thelakkat, and M. Retsch, ‘Plasmonic nanomeshes: their ambivalent role as transparent electrodes in organic solar cells’, *Sci. Rep.*, vol. 7, p. 42530, Feb. 2017, doi: 10.1038/srep42530.

© 2020 by the authors. Submitted for possible open access publication under the terms and conditions of the Creative Commons Attribution (CC BY) license (http://creativecommons.org/licenses/by/4.0/).
